# Serial Analysis of Antimitochondrial Antibody in Patients with Primary Biliary Cirrhosis

**DOI:** 10.1080/10446670410001722113

**Published:** 2004-06

**Authors:** Gordon D. Benson, Kentaro Kikuchi, Hiroshi Miyakawa, Atsushi Tanaka, Mitchell R. Watnik, M. Eric Gershwin

**Affiliations:** 1 Department of Medicine UMDNJ-Robert Wood Johnson Medical School Camden NJ USA; 2 Fourth Department of Internal Medicine Teikyo University School of Medicine Kanagawa Japan; 3 Department of Medicine Teikyo University School of Medicine Tokyo Japan; 4 Division of Rheumatology Allergy and Clinical Immunology University of California at Davis School of Medicine Davis CA USA

## Abstract

Antimitochondrial antibodies (AMAs) are the classic serologic marker in primary biliary cirrhosis (PBC). However, there have been only limited attempts to study changes in titer or isotype analysis of such AMAs in patients followed for long periods of time

We took advantage of stored sera from well-characterized patients with PBC followed for a period of 7-28 years (mean duration of 13.5 years). Immunoblot and enzyme-linked immunosorbant assays were performed against PDC-E2, BCOADC-E2 and OGDC-E2 as well as isotype analysis of antigen-specific IgG, IgA and IgM antibodies against each of these mitochondrial autoantigens. Sera were analyzed for total IgG, IgA and IgM by radial immunodiffusion. The sera titer of AMAs was significantly higher in younger patients with PBC. Indeed, age of onset of clinical PBC was a significant predictor for the highest values of sera AMAs. In contrast, the AMA titer did not significantly change over time in this prolonged longitudinal study. The total sera levels of the individual immunoglobulins did not show a time-dependent change, when based on age of onset of the disease. Higher titers of AMAs were noted in the younger patients. Furthermore, despite this long follow-up, there was no evidence for a significant change in AMA levels; also, levels were not influenced by drug therapy used during the period of observation.


*Clinical & Developmental Immunology*, June 2004, Vol. 11 (2), pp. 129–133

Taylor & Francis healthsciences

### Serial Analysis of Antimitochondrial Antibody in Patients with Primary Biliary Cirrhosis

GORDON D. BENSON[a,*], KENTARO KIKUCHI[b], HIROSHI MIYAKAWA[b], ATSUSHI TANAKA[c], MITCHELL R. WATNIK[d] and M. ERIC GERSHWIN[d]

[a]*Department of Medicine, UMDNJ-Robert Wood Johnson Medical School, 401 Haddon Avenue, Camden, NJ 08103, USA;* [b]*Fourth Department of Internal Medicine, Teikyo University School of Medicine, Kanagawa, Japan;* [c]*Department of Medicine, Teikyo University School of Medicine, Tokyo, Japan;* [d]*Division of Rheumatology, Allergy and Clinical Immunology, University of California at Davis, School of Medicine, Davis, CA, USA*

Antimitochondrial antibodies (AMAs) are the classic serologic marker in primary biliary cirrhosis (PBC). However, there have been only limited attempts to study changes in titer or isotype analysis of such AMAs in patients followed for long periods of time.

We took advantage of stored sera from well-characterized patients with PBC followed for a period of 7–28 years (mean duration of 13.5 years). Immunoblot and enzyme-linked immunosorbant assays were performed against PDC-E2, BCOADC-E2 and OGDC-E2 as well as isotype analysis of antigen-specific IgG, IgA and IgM antibodies against each of these mitochondrial autoantigens. Sera were analyzed for total IgG, IgA and IgM by radial immunodiffusion. The sera titer of AMAs was significantly higher in younger patients with PBC. Indeed, age of onset of clinical PBC was a significant predictor for the highest values of sera AMAs. In contrast, the AMA titer did not significantly change over time in this prolonged longitudinal study. The total sera levels of the individual immunoglobulins did not show a time-dependent change, when based on age of onset of the disease. Higher titers of AMAs were noted in the younger patients. Furthermore, despite this long follow-up, there was no evidence for a significant change in AMA levels; also, levels were not influenced by drug therapy used during the period of observation.

*Keywords*: Antimitochondrial antibodies; Primary biliary cirrhosis; Immunoblot assay; ELISA

### INTRODUCTION

The presence of antimitochondrial antibodies (AMAs) have been known to be associated with primary biliary cirrhosis (PBC) for more than four decades (Mackay, 1958; Walker *et al.*, 1965). The rigorous definition of the antigens recognized by anti-mitochondrial antibodies have helped in our understanding of not only the specificity of these autoantibodies, but also the association of T cell reactivity with the same antigens (Gershwin *et al.*, 1987; Shimoda *et al.*, 1998; Gershwin *et al.*, 2000; Kita *et al.*, 2002a,b). AMAs have become important in the evaluation of patients with chronic liver disease and have provided the impetus for the early diagnosis of PBC (James *et al.*, 1981; Metcalf *et al.*, 1996). There have been a variety of immunological procedures used for their identification, most commonly indirect immunofluorescence (IF), immunoblot assays and enzyme-linked immunosorbant assays (ELISA) (Leung *et al.*, 1997). These assays are of varying sensitivity, specificity and complexity.

The major autoantigens recognized by sera from patients with PBC have been identified as members of the 2-oxo-acid dehydrogenase complex (2-OADC), including the E2 subunit of the pyruvate dehydrogenase complex (PDC-E2), the E2 subunit of the branched chain 2-oxo-acid dehydrogenase complex (BCOADC-E2) and the E2 subunit of the 2-oxo glutarate dehyrogenase complex (OGDC-E2) (Gershwin *et al.*, 1987; Leung and Gershwin, 1989). Dihydrolipoamide dihydrogenase or PDC-E2 is the major autoantigen of PBC and is recognized by 80–90% of sera (Van de Water *et al.*, 1988). In about 60% of PBC patients there is reactivity with BCOADC-E2; some 4–13% will recognize only BCOADC-E2. OGDC-2E is recognized by 30–80% of sera and, rarely, may be the only reaction in established PBC (Miyakawa *et al.*, 2001). Availability of these individual recombinant autoantigens and a triple expression hybrid clone has facilitated development of a highly specific and sensitive ELISA. In a comparison study of 191 patients, 161 or 84% were AMA-positive by IF and 179 or 94% by ELISA using recombinant antigens. Twenty-two (73%) of the 30 patients that were AMA-negative by IF were AMA-positive with this ELISA assay; all controls were negative and there were no false

*Corresponding author. Tel.: +1-856-342-2935. Fax: +1-856-757-9772. E-mail: benson@umdnj.edu

ISSN 1740-2522 print/ISSN 1740-2530 online © 2004 Taylor & Francis Ltd
DOI: 10.1080/10446670410001722113

positives in patients with liver disease (Miyakawa *et al.*, 2001). We chose this latter technology and applied it to a collection of stored sera from PBC patients followed up for a period of 7–28 years. This allowed us to quantitate AMAs over an extended period. In addition, the levels of sera immunoglobulins were compared at the beginning and end of the period of observation.

### MATERIALS AND METHODS

### Patients

Twenty patients with PBC diagnosed with the characteristic clinical and pathological features (Sherlock and Scheuer, 1973; Kaplan, 1987; Neuberger, 1997) were followed for 7–28 years for a mean of 13.5 years. Nineteen were women; age ranged from 25 to 73 years at the time of initial evaluation. During the period of observation, 5 were treated with ursodeoxycholate, 6 with colchicine and 7 with a combination of the two agents. There were no instances of liver failure and none required liver transplantation.

### Sera Immunoglobulins

Immunoglobulins levels were determined by radial immunodiffusion; normal ranges were IgG (820–1740 mg/dl), IgA (90–400 mg/dl) and IgM (52–270 mg/dl).

### Antimitochondrial Antibodies

Immunoblot assays were performed on sera at a dilution of 1:100 utilizing a bovine heart mitochondrial preparation (Miyakawa *et al.*, 1999). Sera were assayed for PDC-E2, BCOADC-E2 and OGDC-E2 utilizing a polyvalent secondary antibody to human Ig and individual antibodies for IgG, IgA and IgM, including monospecific antibodies for IgG class and isotypes, IgG1-4. The unique *Escherichia coli* buffer was used and the cut-off for determining positivity was the mean of the controls + 10 SD. These methods have been previously described (Miyakawa *et al.*, 2001). Similarly, the sera were assayed using the triple expression hybrid clone, MIT3, with a polyvalent secondary antibody to Ig and individual antibodies for IgG, IgA and IgM (Moteki *et al.*, 1996; Miyakawa *et al.*, 2001). In all cases, recombinant autoantigens were used and quality controls, including duplicate assays, were performed; results were appropriately diluted to insure linearity. In addition, the influence of therapy was determined. For analysis of data, a multivariate analysis of variance model (MANOVA) was used, including the outcome variable for immunoglobulin levels and the ELISA values against individual autoantigens and their isotypes. For many of the variables, a transformation was required to satisfy the assumption of normality for the comparisons of the means of the first and last samples from each subject. In presenting the "means" and confidence intervals in the tables, we "back-transformed" those variables to the original scale when a transformation was necessary. The back-transformation prevents us from giving standard errors of the estimates (as well as from interpreting the back-transformed mean as an arithmetic mean). Rather, it is preferable to obtain a confidence interval and back-transform it to give a confidence interval for the population center (interpreted literally, this would be the population median) (Bland and Altman, 1996).

### RESULTS

Eighty samples from these 20 well-characterized patients were tested; one subject was eliminated from the comparison analysis because AMA was considered to be absent at the time of the initial evaluation and continued to be absent during the period of observation. Serum from this patient indicated presence of PDC-E2 with the immunoblot assay but the level failed to exceed the cut-off established for the ELISA. All values for the sera collected at time of diagnosis will be compared to the last sample available from each patient. Table I shows the immunoglobulin levels for IgG, IgA and IgM. The total serum IgG and IgA levels did not change during this extended period of observation. There was a decrease in the mean IgM levels from 368 to 267 mg/dl and a decrease in abnormal levels from 13/19 (68%) to 9/19 (47%) but the changes were not significant. Results of the immunoblot assays are shown in Table II. In sera collected at the time of diagnosis, 18/19 (95%) showed a reaction with PDC-E2, 15/19 (79%) with BCOADC-E2 and 12/19 (63%) with OGDC-E2. Serum from a single patient (5%) reacted only with BCOADC-E2. There were no significant changes between sera collected at the beginning and end of observation. Table III shows the results of the quantitative ELISA for analysis of the three major autoantigens,

TABLE I Levels of sera immunoglobulins

| Ig levels (mg/dl) | First observation Mean | First observation 95% CI | First observation Abnormals | Last observation Mean | Last observation 95% CI | Last observation Abnormals | *p*-Value |
|---|---|---|---|---|---|---|---|
| IgG | 1442 | (1218, 1666) | 3 | 1454 | (1176, 1733) | 4 | 0.893 |
| IgA | 203 | (159, 247) | 1 | 232 | (172, 291) | 2 | 0.162 |
| IgM | 368 | (277, 459) | 13 | 267 | (206, 328) | 9 | 0.071 |

$N = 19$; mean duration of 13.5 years. The total IgG and IgA levels did not change. There was a decrease in IgM levels but the decrease was not significant, although the number of values that exceeded the normal range decreased from 13 to 9.

TABLE II Immunoblotting to recombinant mitochondrial proteins

| | First observation ($N$=19) | Last observation ($N$=19) |
|---|---|---|
| PDC-E2 | | |
| IgG class | 17 | 18 |
| IgA class | 16 | 17 |
| IgM class | 18 | 17 |
| At least 1 of them | 18 | 18 |
| BCOADC-E2 | | |
| IgG class | 14 | 15 |
| IgA class | 8 | 6 |
| IgM class | 15 | 13 |
| At least 1 of them | 15 | 16 |
| OGDC-E2 | | |
| IgG class | 11 | 10 |
| IgA class | 0 | 0 |
| IgM class | 11 | 6 |
| At least 1 of them | 12 | 11 |
| PDC-E2 + BCOADC-E2 + OGDC-E2 | 8 | 10 |
| PDC-E2 + BCOADC-E2 | 5 | 5 |
| PDC-E2 + OGDC-E2 | 4 | 1 |
| PDC-E2 alone | 1 | 2 |
| BCOADC-E2 alone | 1 | 1 |
| OGDC-E2 alone | 0 | 0 |

$N = 19$; mean duration of 13.5 years. In sera collected at the time of diagnosis, 18/19 (95%) showed a reaction with PDC-E2, 15/19 (79%) with BCOADC-E2 and 12/19 (63%) with OGDC-E2. Serum from a single patient (5%) reacted only with BCOADC-E2. There were no significant changes between sera collected at the beginning and end of observation.

PDC-E2, BCOADC-E2 and OGDC-E2, as determined by specific antibodies for class and isotypes. The values were remarkably stable during this extended period of observation but there were a few significant changes, e.g. the anti-IgA antibody to the autoantigen, PDC-E2 showed an increase, whereas the anti-IgG and anti-IgM antibodies to BCOADC-E2 showed a decrease. Further analysis showed that the sera titer of AMAs was significantly higher in younger patients with PBC ($p$-value $= 0.0002$). Indeed, age of onset of PBC posed a significant predictor for the highest values of sera AMAs. Therapy with ursodeoxycholate ($p = 0.687$), colchicine ($p = 0.473$) or the combination ($p = 0.583$) had no effect on the levels. Results of the assays for the AMAs recognizing the recombinant the triple-expression hybrid clone autoantigens, MIT3, are given in Table IV. A polyvalent second antibody to human Ig as well as individual antibodies, anti-IgG, IgA and IgM, were utilized. No significant changes in antibody levels were noted during this period of observation.

### DISCUSSION

Although previous surveys have identified AMA several years before recognition of abnormal liver studies or

TABLE III Quantitative ELISA for the major autoantigens, PDC-E2, BCOADC-E2 and OGDC-E2

| | First observation mean 95% CI | Last observation mean 95% CI | $p$-Value |
|---|---|---|---|
| *PDC-E2 ELISA* | | | |
| IgG | 0.728 (0.422, 1.035) | 0.779 (0.489, 1.069) | 0.531 |
| Isotypes: | | | |
| G1 | 0.030 (0.025, 0.038) | 0.029 (0.026, 0.033) | 0.706 |
| G2 | 0.180 (0.101, 0.319) | 0.221 (0.117, 0.418) | 0.416 |
| G3 | 0.631 (0.277, 0.984) | 0.780 (0.317, 1.243) | 0.443 |
| G4 | 0.109 (0.078, 0.139) | 0.112 (0.080, 0.143) | 0.885 |
| IgA | 0.043 (0.026, 0.071) | 0.061 (0.038, 0.100) | 0.013 |
| IgM | 0.183 (0.113, 0.295) | 0.188 (0.115, 0.306) | 0.897 |
| *BCOADC-E2 ELISA* | | | |
| IgG | 0.117 (0.044, 0.310) | 0.082 (0.032, 0.212) | 0.022 |
| Isotypes: | | | |
| G1 | 0.059 (0.054, 0.065) | 0.052 (0.048, 0.058) | 0.077 |
| G2 | 0.127 (0.092, 0.203) | 0.109 (0.081, 0.165) | 0.169 |
| G3 | 0.095 (0.060, 0.151) | 0.099 (0.051, 0.191) | 0.875 |
| G4 | 0.103 (0.076, 0.139) | 0.100 (0.075, 0.134) | 0.755 |
| IgA | 0.022 (0.018, 0.028) | 0.024 (0.018, 0.032) | 0.389 |
| IgM | 0.138 (0.076, 0.248) | 0.073 (0.033, 0.159) | 0.047 |
| *OGDC-E2 ELISA* | | | |
| IgG | 0.051 (0.023, 0.115) | 0.050 (0.024, 0.106) | 0.849 |
| Isotypes: | | | |
| G1 | 0.055 (0.046, 0.066) | 0.053 (0.047, 0.060) | 0.775 |
| G2 | 0.089 (0.074, 0.110) | 0.092 (0.076, 0.118) | 0.589 |
| G3 | 0.039 (0.027, 0.055) | 0.045 (0.029, 0.069) | 0.422 |
| G4 | 0.057 (0.048, 0.067) | 0.053 (0.045, 0.063) | 0.528 |
| IgA | 0.068 (0.017, 0.119) | 0.052 (0.027, 0.077) | 0.324 |
| IgM | 0.047 (0.025, 0.091) | 0.400 (0.020, 0.080) | 0.327 |

The values were remarkably stable during this extended period of observation but there were a few significant changes, e.g. the anti-IgA antibody to the autoantigen, PDC-E2 showed an increase, whereas the anti-IgG and anti-IgM antibodies to BCOADC-E2 showed a decrease.

TABLE IV AMAs against the recombinant triple expression hybrid clone autoantigens, MIT3

| | First observation (*N*=19) Mean 95% CI | Last observation (*N*=19) Mean 95% CI | *p*-Value |
|---|---|---|---|
| MIT-3 ELISA | | | |
| IgG + A + M | 2.418 (1.825, 3.011) | 2.320 (1.749, 2.891) | 0.483 |
| IgG class | 1.810 (1.438, 2.181) | 1.790 (1.409, 2.170) | 0.850 |
| IgA class | 0.150 (0.078, 0.288) | 0.134 (0.070, 0.254) | 0.528 |
| IgM class | 0.566 (0.331, 0.801) | 0.526 (0.256, 0.796) | 0.710 |

No significant changes in antibody levels were noted during this period of observation.

clinical evidence of PBC (Walker *et al.*, 1965, 1970; James *et al.*, 1981), there is scant information concerning autoantibody levels during the clinical course of the disease. The fact that 95% of patients with PBC are now recognized to have AMA with current methodology, suggests that the majority have had the antibody for years before laboratory or clinical features are recognized. Indeed, it seems unlikely that the patient with PBC who is truly negative, is likely to become positive once the clinical diagnosis is established on laboratory or histological changes. The persistence of stable antibody levels during the course of the illness, suggests that there is either a continued immunological insult that perpetuates production of AMA or, alternatively, the AMA at the time of disease recognition is already a long-established antibody activity following a potent antigenic stimulus.

These results suggest that the AMA response was established long before the clinical diagnosis of PBC. In fact, random surveys of large numbers of healthy subjects have disclosed the presence of AMAs for several years before clinical diagnosis (Mattalia *et al.*, 1998; Gershwin *et al.*, 2000). Thus, it is not surprising that the AMA titer does not change over time to a significant extent. An analogy can be made to anti-tetanus and diphtheria responses, both of whom will continue with high titer for several decades following the last immunization. In fact, this data is also consistent with the relatively small change which occurs in AMA titer following liver transplantation (Van de Water *et al.*, 1996; Luettig *et al.*, 1998).

This study was valuable because of the characteristics of the sera from the same patients followed for this unique period of time. Although these patients may not be entirely representative of the spectrum of disease observed in PBC, they do provide an important framework and the data should be discussed with relation to similar observations. For example, changes in immunoglobulin levels have long been noted in association with chronic liver disease (Eliakim *et al.*, 1972). In PBC, IgM has been found to be increased (Paronetto *et al.*, 1964; Bevan, 1966; Feizi, 1968; Walker and Doniach, 1968), an increase which may be supportive of the diagnosis when AMA is absent or present in low titer (Bevan *et al.*, 1969). Immunoglobulin M was elevated in 13/19 (68%) at the time of diagnosis in this group. Although the abnormal levels decreased to 9/19 (47%) during observation, the decrease was not statistically significant.

Reactivity to the three recombinant mitochondrial proteins (Table II) are similar to other studies, in that sera recognized 18/19 (95%) of PDC-E2, 15/19 (79%) of BCOADC-E2 and 12/19 (63%) of OGDC-E2 (Miyakawa *et al.*, 2001). A single serum recognized only BCOADC-E2. The patterns of reactivity were remarkably durable and there were only insignificant changes. The results of a detailed analysis utilizing the three major autoantigens, PDC-E2, BCOADC-E2 and OGDC-E2 and the second antibodies, IgG, IgA and IgM, plus IgG isotypes, G1-4, are given in Table III. Again, the autoantibodies levels were remarkably stable. The only significant changes included an increase in IgA reactivity to PDC-E2 and a decrease in IgG and IgM reactivity to BCOADC-E2. Interestingly, the levels of AMA were significantly higher in younger patients at the time of diagnosis. Indeed, age of onset of PBC was a predictor for the highest AMA levels. There were no significant changes in the AMA levels when the MIT3 triple-expression hybrid autoantigen was used. The epitope recognized by antimitochondrial antibodies is similar to the region recognized by autoreactive $CD4^+$ and CD8 T cells. More importantly, the T cell precursor frequency in liver against these autoepitopes is up to 100 fold higher than it is in blood, suggesting that the antimitochondrial response is either directly related to disease pathogenesis, or else intimately related to disease initiation (Shimoda *et al.*, 1998; Kita *et al.*, 2002a,b). We suggest that future studies focus on the most recently diagnosed patients with PBC and/or perhaps be able to screen and identify sera from asymptomatic patients in hopes of identifying the initiating event.

### *References*

Bevan, G. (1966) "Primary biliary cirrhosis-positive antibody tests associated with increased immunoglobulin", *Proc. R. Soc. Med.* **59**, 567–568.

Bevan, G., Baldus, W.P. and Gleich, G.J. (1969) "Serum immunoglobulin levels in cholestasis", *Gastroenterology* **56**, 1040–1046.

Bland, J.M. and Altman, D.G. (1996) "Transformations, means, and confidence intervals", *Br. Med. J.* **312**, 1079.

Eliakim, M., Zlotnick, A. and Slavin, S. (1972) "Gammopathy in liver disease", *Prog. Liver Dis.* **4**, 403–417.

Feizi, T. (1968) "Immunoglobulins in chronic liver disease", *Gut* **9**, 193–198.

Gershwin, M.E., Mackay, I.R., Sturgess, A. and Coppel, R.L. (1987) "Identification and specificity of a cDNA encoding the 70 kd mitochondrial antigen recognized in primary biliary cirrhosis", *J. Immunol.* **138**, 3525–3531.

Gershwin, M.E., Ansari, A.A., Mackay, I.R., *et al.* (2000) "Primary biliary cirrhosis: an orchestrated immune response against epithelial cells", *Immunol. Rev.* **174**, 210–225.

James, O., Macklon, A.F. and Watson, A.J. (1981) "Primary biliary cirrhosis—a revised clinical spectrum", *Lancet* **1**, 1278–1281.

Kaplan, M.M. (1987) "Primary biliary cirrhosis", *N. Engl. J. Med.* **316**, 521–528.

Kita, H., Matsumura, S., He, X.S., *et al.* (2002a) "Quantitative and functional analysis of PDC-E2-specific autoreactive cytotoxic T lymphocytes in primary biliary cirrhosis", *J. Clin. Investig.* **109**, 1231–1240.

Kita, H., Lian, Z.X., Van de Water, J., *et al.* (2002b) "Identification of HLA-A2-restricted CD8(+) cytotoxic T cell responses in primary biliary cirrhosis: T cell activation is augmented by immune complexes cross-presented by dendritic cells", *J. Exp. Med.* **195**, 113–123.

Leung, P.S. and Gershwin, M.E. (1989) "The molecular structure of autoantigens", *Curr. Opin. Immunol.* **2**, 567–575.

Leung, P.S., Coppel, R.L., Ansari, A., *et al.* (1997) "Antimitochondrial antibodies in primary biliary cirrhosis", *Semin. Liver Dis.* **17**, 61–69.

Luettig, B., Boeker, K.H., Schoessler, W., *et al.* (1998) "The antinuclear autoantibodies Sp100 and gp210 persist after orthotopic liver transplantation in patients with primary biliary cirrhosis", *J. Hepatol.* **28**, 824–828.

Mackay, I.R. (1958) "Primary biliary cirrhosis showing a high titer of autoantibody; report of a case", *N. Engl. J. Med.* **258**, 185–188.

Mattalia, A., Quaranta, S., Leung, P.S., *et al.* (1998) "Characterization of antimitochondrial antibodies in health adults", *Hepatology* **27**, 656–661.

Metcalf, J.V., Mitchison, H.C., Palmer, J.M., *et al.* (1996) "Natural history of early primary biliary cirrhosis", *Lancet* **348**, 1399–1402.

Miyakawa, H., Kawaguchi, N. and Abe, K. (1999) "Serial changes of serum anti-M2 proteins in patients with primary biliary cirrhosis: a follow-up study by immunoblotting", *Hepatol. Res.* **13**, 143–152.

Miyakawa, H., Tanaka, A., Kikuchi, K., *et al.* (2001) "Detection of antimitochondrial autoantibodies in immunofluorescent AMA-negative patients with primary biliary cirrhosis using recombinant autoantigens", *Hepatology* **34**, 243–248.

Moteki, S., Leung, P.S., Coppel, R.L., *et al.* (1996) "Use of a designer triple expression hybrid clone for three different lipoyl domain for the detection of antimitochondrial autoantibodies", *Hepatology* **24**, 97–103.

Neuberger, J. (1997) "Primary biliary cirrhosis", *Lancet* **350**, 875–879.

Paronetto, F., Schaffner, F. and Popper, H. (1964) "Immunocytochemical and serologic observations in primary biliary cirrhosis", *N. Engl. J. Med.* **271**, 1123–1128.

Sherlock, S. and Scheuer, P.J. (1973) "The presentation and diagnosis of 100 patients with primary biliary cirrhosis", *N. Engl. J. Med.* **289**, 674–678.

Shimoda, S., Van de Water, J., Ansari, A., *et al.* (1998) "Identification and precursor frequency analysis of a common T cell epitope motif in mitochondrial autoantigens in primary biliary cirrhosis", *J. Clin. Investig.* **102**, 1831–1840.

Van de Water, J., Fregeau, D., Davis, P., *et al.* (1988) "Autoantibodies of primary biliary cirrhosis recognize dihydrolipoamide acetyltransferase and inhibit enzyme function", *J. Immunol.* **141**, 2321–2324.

Van de Water, J., Gerson, L.B., Ferrell, L.D., *et al.* (1996) "Immunohistochemical evidence of disease recurrence after liver transplantation for primary biliary cirrhosis", *Hepatology* **24**, 1079–1084.

Walker, G. and Doniach, D. (1968) "Antibodies and immunoglobulins in liver disease", *Gut* **9**, 266–269.

Walker, J.G., Doniach, D., Roitt, I.M. and Sherlock, S. (1965) "Serological tests in diagnosis of primary biliary cirrhosis", *Lancet* **39**, 827–831.

Walker, J.G., Doniach, D. and Doniach, I. (1970) "Mitochondrial antibodies and subclinical liver disease", *Q. J. Med.* **39**, 31–48.

